# Genome-wide transcriptional regulation of estrogen receptor targets in fallopian tube cells and the role of selective estrogen receptor modulators

**DOI:** 10.1186/s13048-016-0213-3

**Published:** 2016-02-15

**Authors:** Georgette Moyle-Heyrman, Matthew J. Schipma, Matthew Dean, David A. Davis, Joanna E. Burdette

**Affiliations:** Department of Medicinal Chemistry and Pharmacognosy, University of Illinois at Chicago, Chicago, Illinois 60607 USA; Department of Human Biology, University of Wisconsin – Green Bay, Green Bay, Wisconsin 54311 USA; Next Generation Sequencing Core Facility, Feinberg School of Medicine, Northwestern University, Chicago, Illinois 60611 USA

**Keywords:** Estrogen, Fallopian tube epithelium, High-grade serous cancer, Ovarian cancer, Selective estrogen receptor modulators

## Abstract

**Background:**

The fallopian tube epithelium is one of the potential sources of high-grade serous ovarian cancer (HGSC). The use of estrogen only hormone replacement therapy increases ovarian cancer (OVCA) risk. Despite estrogen’s influence in OVCA, selective estrogen receptor modulators (SERMs) typically demonstrate only a 20 % response rate. This low response could be due to a variety of factors including the loss of estrogen receptor signaling or the role of estrogen in different potential cell types of origin. The response of fallopian tube epithelium to SERMs is not known, and would be useful when determining therapeutic options for tumors arising from this cell type, such as HGSC.

**Results:**

Using normal murine derived oviductal epithelial cells (mouse equivalent to the fallopian tube) estrogen receptor expression was confirmed and interaction with its ligand, estradiol, triggered mRNA and protein induction of progesterone receptor (PR). The SERMs 4-hydroxytamoxifen, raloxifene and desmethylarzoxifene, functioned as estrogen receptor antagonists in oviductal cells. Cellular proliferation and migration assays suggested that estradiol does not significantly impact cellular migration and increased proliferation. Further, using RNAseq, the oviduct specific transcriptional genes targets of ER when stimulated by estradiol and 4-hydroxytamoxifen signaling were determined and validated. The RNA-seq revealed enrichment in proliferation, anti-apoptosis, calcium signaling and steroid signaling processes. Finally, the ER and PR receptor status of a panel of HGSC cell lines was investigated including Kuramochi, OVSAHO, OVKATE, OVCAR3, and OVCAR4. OVSAHO demonstrated receptor expression and response, which highlights the need for additional models of ovarian cancer that are estrogen responsive.

**Conclusions:**

Overall, the fallopian tube has specific gene targets of estrogen receptor and demonstrates a tissue specific response to SERMs consistent with antagonistic action.

**Electronic supplementary material:**

The online version of this article (doi:10.1186/s13048-016-0213-3) contains supplementary material, which is available to authorized users.

## Background

Ovarian cancer (OVCA) is the most lethal gynecological malignancy and fifth leading cause of cancer-related death in women [1]. The fallopian tube epithelium (referred to as oviduct epithelium in all species except primates) is one of the likely progenitor cell types for the most common and deadly OVCA histotype, high-grade serous cancer (HGSC) with the alternative cellular source being the ovarian surface epithelium (OSE) [2]. Morphological, immunological, and gene expression analysis of HGSC tumors, suggest a close relationship to fallopian tube epithelium rather than OSE [3, 4]. Precursor lesions, termed the p53 signature, have been identified in the fimbriated end of the fallopian tube [5]. In cases of HGSC, these p53 signatures in the fallopian tube had the same mutation as their corresponding ovarian carcinoma [5]. Additional precursor lesions termed serous tubal intraepithelial carcinomas (STICs) have been identified in the fallopian tubes (but not ovaries) of women undergoing risk-reducing salpingo-oophorectomy, particularly in women with *BRCA*1/2 mutations [6]. Furthermore, mouse models of HGSC have been produced by inducing BRCA, PTEN, and p53 mutation in the oviductal epithelium [7].

Risk factors for non-heritable OVCA include null parity, infertility, the number of lifetime ovulations and the use of estrogen only-hormone replacement therapy [8]. Estrogen is a steroid hormone that functions in multiple tissues in the body, including the fallopian tube epithelium. While the reproductive role of estrogen in the fallopian tube is to facilitate movement and maturation of eggs, sperm and fertilized embryos between the ovary and uterus [9], the function in terms of tumor initiation and progression is not clear. Estrogen signals in the cell through three main receptors. Estrogen receptor α (ERα) and ERβ are ligand activated transcription factors [10]. G protein coupled receptor (GPER) is a membrane bound factor that signals through a non-genomic mechanism [11]. Given the recent findings suggesting that OVCA is increased by hormone replacement therapy containing estrogen and the fallopian tube may be the source of HGSC, the estrogen receptor targets in the fallopian tube should be defined [12].

ERα and a gene target of estrogen signaling, progesterone receptor (PR), are prognostic biomarkers in OVCA [13]. ERα is expressed in 80 % of HGSC, but PR is expressed in only 31 % [13]. Successful treatment of OVCA with selective estrogen receptor modulators (SERM) therapy has been limited [14]. SERMs are ER ligands that function as either agonists or antagonists in a cell type specific manner [15]. Given that HGSC may arise from the fallopian tube, understanding the response of normal fallopian tube epithelium to estrogen and SERMS is important for understanding the implications of SERM therapy on OVCA risk.

Murine oviduct epithelial (MOE) cells were utilized to investigate estrogen signaling in a putative HGSC precursor. MOE cells are estrogen responsive and the SERMs 4-hydroxytamoxifen (4OHT), raloxifene (RAL) and desmethylarzoxifene (DMA) antagonize 17-βestradiol (E_2_) in this cell type. The MOE specific transcriptional targets of estrogen signaling were determined by RNAseq. Finally, the receptor status for ERα, ERβ and PR was determined in a panel of HGSC cell lines. Our results highlight the need to consider the E_2_ response of putative progenitor cell populations of HGSC to investigate estrogen’s role in initiation and progression of OVCA. The occurrence and survival rates for OVCA have not improved in over 40 years emphasizing the necessity for better understanding and effectively treating or preventing this deadly disease. This study demonstrates that estrogen receptor activates unique oviduct-specific targets that may provide new targets for preventing or treating fallopian tube derived tumors.

## Methods

### Cell culture

MOE and MOSE cells (passages 7-25) were cultured as previously described [16, 17]. For experiments investigating E_2_ and SERM response, cells were cultured at least 48 h in “stripped media” consisting of phenol red free α-modified Eagle’s medium (Life Technologies, Carlsbad, CA) supplemented with 10 % v/v charcoal stripped fetal bovine serum (FBS) (Life Technologies) [18], 1 mg/mL gentamycin (Mediatech, Manassas, VA), 2 mM L-glutamine (Life Technologies), 100 U/mL penicillin and 50 μg/mL streptomycin (Roche, Indianapolis, IN) prior to treatments. KURAMOCHI (passages 15-25), OVSAHO (passages 45-60) and OVKATE (passages 45-60) cell lines were purchased from the JCRB Cell bank and maintained in RPMI1640 (Mediatech) supplemented with 10 % FBS,100 U/mL penicillin and 50 μg/mL streptomycin. OVSAHO were cultured for 48 hours (h) prior to treatment and treated in phenol-red free RPMI1640 (Life Technologies) supplemented with 10 % charcoal stripped FBS, 100 U/mL penicillin and 50 μg/mL streptomycin. OVCAR4 (passages 15-30) were acquired from the National Cancer Institute Division of Cancer Treatment and Diagnosis Tumor Repository and maintained in the same media as KURAMOCHI plus 1 % ι-glutamine. SKOV3 (passages 10-30) and OVCAR3 (passages 3-30) cell lines were acquired from ATCC (Manassas, VA). SKOV3 were cultured in McCoy’s 5A supplemented with 2.3 g/L sodium carbonate, 10 % FBS, 100 U/mL penicillin and 50 μg/mL streptomycin. OVCAR3 were maintained in minimum essential media supplemented with 20 % FBS, 1 % ι-glutamine, 1 % non-essential amino acids, 1 % sodium pyruvate, 100 U/mL penicillin and 50 μg/mL streptomycin. Cell lines were authenticated by STR analysis at DDC Medical (murine) or UIC DNA Services Facility (human).

### Western blotting

Cells were plated at a density of 10-30 × 10^4^ cells per well in a 6-well dish in stripped media. Twenty-four hours post plating cells were washed with PBS and the media was replenished. After another 24 h, cells were washed with PBS and treated with DMSO (0.1 %) or compounds for indicated times.

Following treatment, cells were washed with PBS then harvested on ice in RIPA buffer (50 mM Tris, pH 7.6, 150 mM NaCl, 1 % v/v Triton X-100, 0.1 % w/v sodium dodecyl sulfate) and frozen at -80 °C. Lysates were centrifuged at 14,000 rpm for 5 min and clarified samples were quantified by BCA (Pierce, Rockford, IL), separated by SDS PAGE (8 %) at 100 V for 2.5 h then transferred for 2 h at 25 V to nitrocellulose (GE Healthcare Bio-Sciences, Pittsburgh, PA). Blots were blocked in 5 % milk TBS-T (Tris Buffered Saline-Tween 20) for 1 h at room temperature (RT) followed by overnight incubation at 4 °C in primary antibodies: Anti-ERα (1:300, MC-20 or 1:200 HC-20, Santa Cruz, Dallas, TX), Anti-PR (1:500 H-190, Santa Cruz), Anti-actin (1:1000, Sigma-Aldrich). Following 3 washes in TBS-T, blots were incubated in Anti-rabbit horseradish peroxidase-conjugated secondary (1:1000, Cell Signaling, Cambridge, MA) for 30 min at RT. After secondary, blots were washed 3X in TBS-T then bands were imaged using SuperSignal West Femto Chemiluminescent Substrate (Pierce) on a FluorChem E Imager (ProteinSimple, Santa Clara, CA). Densitometry was performed using Protein Simple software.

### qPCR

Cells were treated as described for Western blots, then harvested in 500 μL of Trizol Reagent (Life Technologies) and frozen at -80 until processed according to manufacturer’s protocol. Purified RNA was treated with DNAseI (New England Biolabs, Ipswich, MA) for 10 min, followed by heat inactivation. 1 μg of purified RNA was reverse transcribed using Revertaid Reverse Transcriptase (Thermo Fisher Scientific, Waltham, MA) in the presence of Ribolock RNAse Inhibitor (Thermo Fisher Scientific). Expression was monitored using Faststart Universal Sybr Green (Rox) (Roche) using protocol: 10 min at 94 °C, 40 cycles of 10 sec at 94 °C, 30 sec at 60 °C followed by a melt curve. Primers listed in Additional file 1: Table S1.

### Immunofluorescence

MOE cells were cultured in charcoal stripped media for 72 h and then plated (50,000 per well) onto Millicell EZ slides (Millipore, Billerica, MA). The next day the cells were fixed with 4 % paraformaldehyde and probed with primary antibody against estrogen receptor α (1:200, MC-20, Santa Cruz) with 10 % goat serum. Slides washed and probed with secondary antibody (AlexaFluro 594 A11037, Life Technologies) before mounting with DAPI containing mounting media (H-1500, Vector Laboratories, Burlingame, CA). Images were captured with a Nikon Eclipse E600 microscope at 40x.

### Proliferation assay

Cells were cultured in stripped media for 72 h followed by passaging and plating of 1,000 cells/well in a 96 well plate. Twenty-four hours post plating, cells were treated with solvent or compounds for 72 h then fixed with 20 % w/v Trichloroacetic acid for 24 h at 4 °C and processed for sulforhodamine B colorimetric assay as described [19]. Absorbance at 505 nm was measured using a BioTek Synergy MX microplate reader (BioTek, Winooski, VT).

### Wound healing assay

Cells were cultured for 72 h as described in the proliferation assay, followed by passaging and plating of 6×10^4^ per well in a 24 well plate. Twenty-four hours post plating a pipette tip induced a scratch across the monolayer of cells. Images of the wound were taken at time zero and 24 h post scratch. The percent closure of the wound was calculated as the area of the scratch at 24 h post scratch divided by the area of the scratch at time zero using ImageJ (National Institutes of Health).

### Luciferase assay

SKOV3 cells were trypsinized and plated in 24-well plate (3.5 × 10^4^ cells/ well) in stripped media. Incubation of cells with pERE-luciferase plasmid (100 ng/well) [20], RSV-β-galactosidase (100 ng/well, [21], and TransIT LT1 transfection reagent (1 μL per well, Mirus Bio, Madison, WI) was performed overnight in fresh media then treated for 24 h. Luciferase production and β-galactosidase activity (for transfection normalization) were measured as described previously [21].

### RNAseq library construction and sequencing

Cells were treated as described in qPCR assay, followed by RNA isolation using Qiagen Qiashredder column, on column DNAse treatment and Qiagen RNAeasy spin columns (Qiagen, Valencia, CA). Library construction and sequencing were performed at the Genomics Core facility at the University of Chicago. RNA quality and quantity were determined with the Agilent Bioanalyzer 2100, with RNA integrity numbers (RIN) of 10 and quantities of 100 ng or more per sample. Samples were enriched for mRNA using oligo-dT columns. Directional 50 bp single-end mRNA libraries were prepared using Illumina TruSeq mRNA Sample Preparation Kits per manufacturer’s instructions. Briefly, polyadenylated mRNAs were captured from total RNA using oligo-dT selection. Next, samples were converted to cDNA by reverse transcription, and each sample was ligated to Illumina sequencing adapters containing unique barcode sequences. Barcoded samples were then amplified by PCR and the resulting cDNA libraries quantified using qPCR. Finally, equimolar concentrations of each cDNA library were pooled and sequenced on the Illumina HiSeq2500.

### Transcriptome analysis

The quality of DNA reads, in fastq format, was evaluated using FastQC. Adapters were removed and reads of poor quality filtered. The data was processed largely following the procedure described in [22]. Briefly, reads were aligned to the *Mus musculus* genome (mm10) using TopHat (v2.0.8b). Subsequently, aligned reads, in conjunction with a gene annotation file for mm10 obtained from the UCSC website, were used to determine the expression of known genes using Cufflinks (v2.1.1). Individual transcript files generated by Cufflinks for each sample were merged into a single gene annotation file, which was then used to perform a differential expression analysis with the Cufflinks routine, cuffdiff. Differential expression was determined by cuffdiff using the procedure described in Trapnell et al [22], using an FDR cutoff value of 0.05. Results of the differential expression analysis were processed with cummeRbund. Differentially expressed genes were separated into upregulated and downregulated lists. A pathway analysis was performed on both gene lists using GeneCoDis [23–25] to identify pathways enriched with genes that were upregulated and downregulated.

### Statistical analysis

Data shown are represented as the mean of at least three experiments, with errors bars representing the standard error. Statistical analysis was conducted with GraphPad Prism (GraphPad, La Jolla, CA) using one-way ANOVA with a Tukey’s post hoc test.

## Results

### Putative OVCA progenitor cell type estrogen responsive

**Fig. 1:**
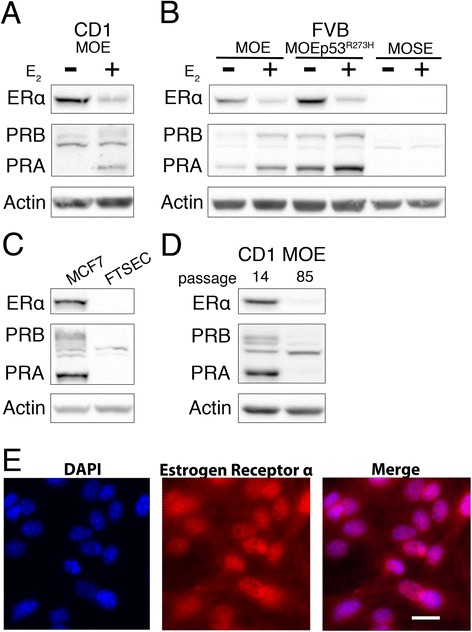
Receptor status and estrogen responsiveness monitored by Western blot analysis. **a** Analysis of ERα and PR expression in response to 24 h 17β-estradiol (1nM, E_2_) treatment in CD1 MOE cells or (**b**) FVB MOE and MOSE cells. **c** Western blot analysis of human fallopian tube secretory epithelial cells (FTSEC) and receptor positive MCF7 breast cancer cells. **d** Receptor protein levels of early passage (P14) and late passage (P85) Cd1 MOE cells. **e** Immunofluorescence in FVB MOE cells for ERα and DAPI counterstain. Scale bar = 20 μm

The fallopian tube (oviduct in the mouse) epithelium is likely one of the sources of HGSC. To investigate the role of estrogen signaling in this precursor cell type of HGSC, we evaluated the response of murine oviductal epithelium (MOE) cells derived from CD1 and FVB murine backgrounds subjected to 17-beta-estradiol (E_2_) treatment (Fig. 1a, b). CD1 MOE cells are a polyclonal cell line consisting of both secretory and ciliated oviductal epithelial cells [16]. The FVB MOE cells are monoclonal, comprised exclusively of secretory oviductal epithelial cells [17]. The disappearance of ERα via proteasome–mediated proteolysis [26], and upregulation of the canonical ER regulated target progesterone receptor (PRA and PRB, two isoforms encoded by the *Pgr* gene) were monitored for E_2_ responsiveness via Western blot analysis. Immunofluorescence revealed that 100 % of FVB MOE cells expressed ERα (Fig. 1e). MOE cell lines demonstrated robust E_2_ responsiveness for these endpoints.

HGSC is a heterogeneous disease, the only common alteration (<96 % of cases) being a mutation in the *Tp53* gene [27]. Intriguingly, FVB MOE cells stably transfected with a plasmid encoding the human *Tp53* gene mutated at R273H [17] expressed elevated protein levels of both ERα and PRA/PRB (Fig. 1b), although the transcriptional strength of PR induction by E_2_ was not significantly different than observed in wildtype MOE FVB cells (Additional file 2: Figure S1a-c).

A human fallopian tube secretory epithelial cell (FTSEC) line [28] did not express detectable ERα and PR, precluding study of E_2_ responsiveness in human cells (Fig. 1c), although transient transfection of a plasmid encoding ERα did recover the ability for E_2_ to induce transcription of *Pgr* (data not shown). Continuous culturing of the CD1 MOE cell line resulted in a decrease of the receptors (Fig. 1d) suggesting growth on plastic is capable of inducing receptor loss. These results were similar to a baboon FTSEC that also lost receptor in culture that could be reactivated [20].

The E_2_ responsiveness of the classically studied OVCA precursor, the ovarian surface epithelium (OSE) was also investigated. A murine ovarian surface epithelium (MOSE) cell line from the FVB background [16], expressed much less ERα and PRA/PRB compared to the MOE cell lines, and further PRA/PRB levels were not altered by E_2_ treatment (Fig. 1b). OSE are known to express the receptors in vivo [29], once again indicating culturing on tissue culture plastic could lead to receptor depletion. Attempts to enhance receptor expression and E_2_ responsiveness of MOSE cells using an HDAC inhibitor (HD13) [30] or the demethyltransferase inhibitor, 5-azacytidine were unsuccessful (Additional file 2: Figure S1d and data not shown).

### SERMs antagonize E_2_ in MOE

**Fig. 2:**
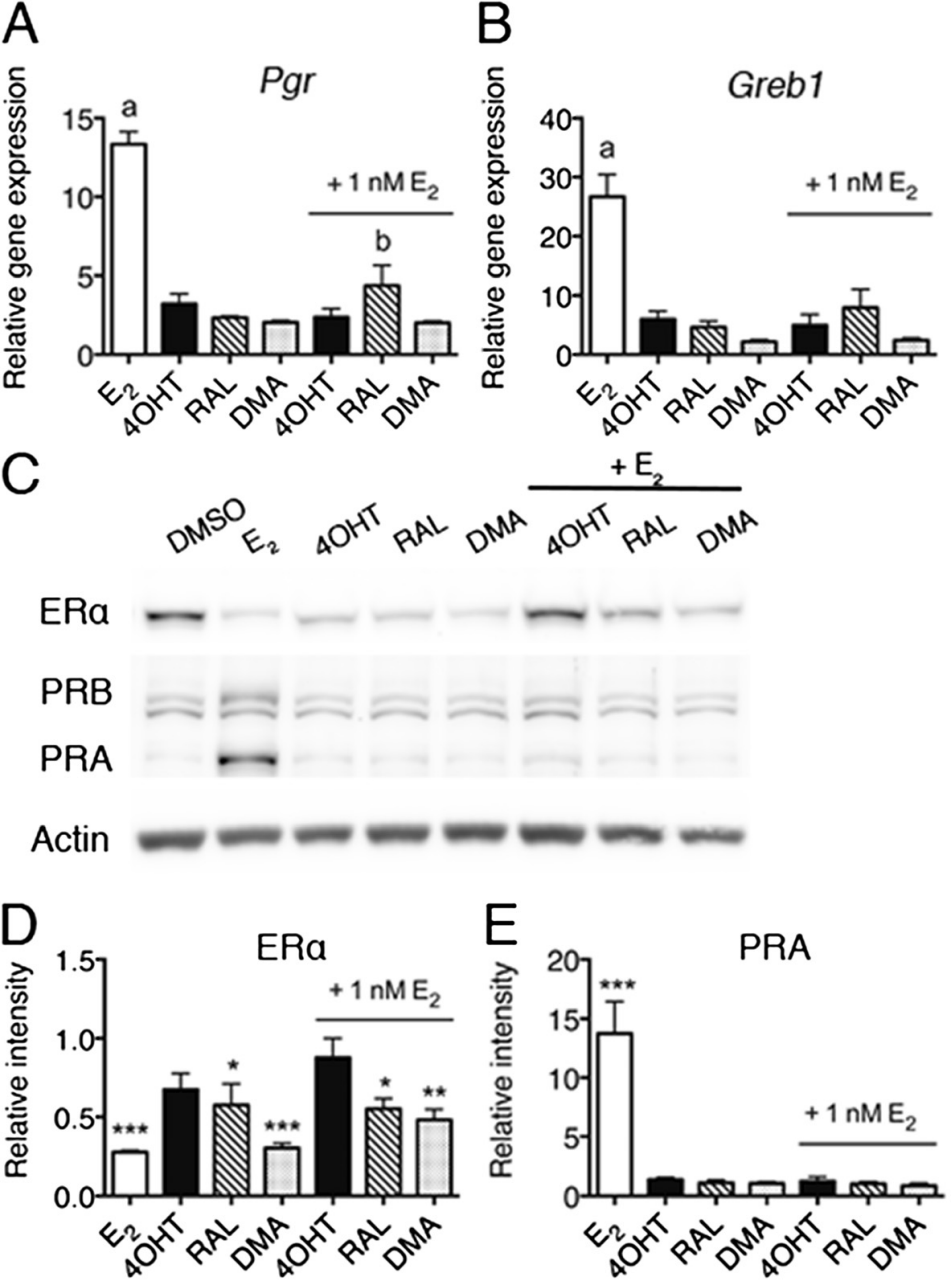
SERMs antagonize E_2_ in CD1 MOE. **a**, **b** qPCR analysis of (**a**) *Pgr* and (**b**) *Greb1* induction in response to 48 h hormone starvation and 24 h treatment with DMSO, 1 nM E_2_ or 100 nM SERMs. “a” indicates significant difference compared to all treatments (*p* < 0.001) and “b” is significantly different than DMSO control treatment (*p* < 0.01). **c** Western blot analysis of CD1 MOE treated for 24 h with 1 nM E_2_ and 100 nM SERMs and the combination. **d**, **e** Densitometry of ERα and PRA bands relative to solvent treated cells normalized to actin. ERα is significantly lower in E_2_, RAL and DMA treated cells, while 4OHT does not result in ERα degradation. PR is significantly upregulated in E_2_ treated cells compared to all conditions. Significance determined by one-way ANOVA followed by Tukey’s post hoc test where * indicates *p* < 0.05, ** indicates *p* < 0.01, *** indicates *p* < 0.001

Women at high risk for breast cancer and OVCA, having a mutation in either BRCA1 or BRCA2 genes, may prophylactically take selective estrogen receptor modulators (SERMs), such as 4-hydroxytamoxifen (4OHT), to decrease their risk of developing breast cancer [31]. SERM action as an ER agonist or antagonist is cell type specific and the effect on ER and the response of the fallopian tube epithelium in terms of cancer biology to SERMs is not well documented. MOE cells were used to elucidate whether the SERMs 4OHT, raloxifene (RAL) and desmethylarzoxifene (DMA), function as ER antagonists or agonists. QPCR analysis of the genes *Pgr* and *Greb1*, revealed significant (*p* < 0.001) induction in response to E_2_ in both CD1 and FVB MOE cells (Fig. 2a, b and Additional file 3: Figure S2a,b). All SERMs tested did not significantly activate *Pgr* or *Greb1* compared to the DMSO but did significantly (*p* < 0.001) antagonize E_2_ dependent induction in both MOE cell lines (Fig. 2a, b and Additional file 3: Figure S2a,b). Western blot analysis of CD1 MOE treated with E_2_ and SERMs revealed significant (*P* < 0.001) PR induction and disappearance of ERα in response to E_2_ treatment (Fig. 2c, d, e). These findings indicate that the SERMs antagonize E_2_ in oviduct epithelial cells.

**Fig. 3:**
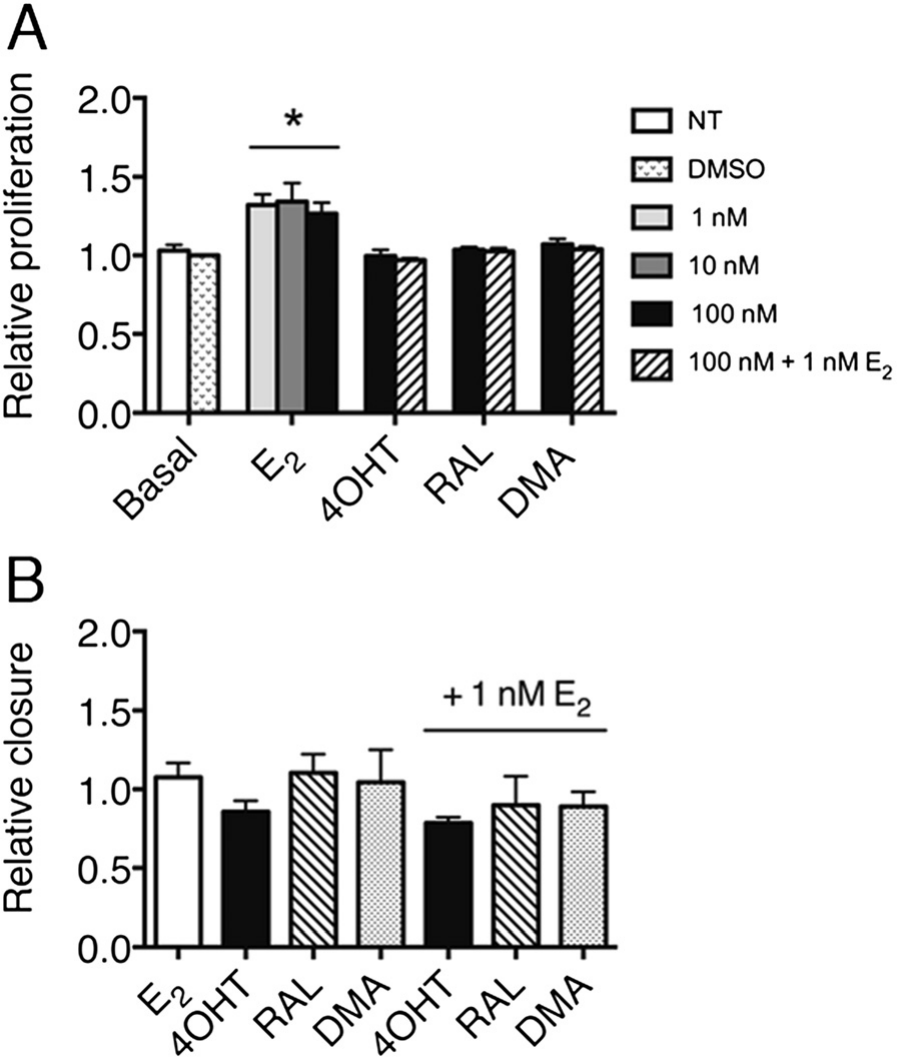
Functional response of CD1 MOE to E_2_ signaling (**a**) Relative growth of CD1 MOE following 72-hour hormone starvation and 72-hour treatment with DMSO, 1 nM E_2_, 100 nM SERMS and the combination as monitored by SRB assay. * indicates significant difference from all other treatments (*p* < 0.05). **b** Relative migration of CD1 MOE cells following 72-hour hormone starvation and 24-hour treatment (wound initiated at time of treatment) with DMSO, 1 nM E_2_ or 100 nM SERMs

E_2_ increases the growth rate in a subset of ER positive OVCA cell lines [32, 33]. The reported functional response of fallopian tube epithelial cells to estrogen signaling involves triggering the differentiation of epithelial cells, protein secretion, and regulation of cilia beating [34]. In vivo proliferation rates have not been reported to change due to ovulation, which has high levels of estrogen in the follicular phase of the cycle. To evaluate the functional response of MOE cells to E_2_ and SERMs, a proliferation assay was employed. Following 72-hour treatment, a small but significant (*p* < 0.05) proliferation increase was observed in the presence of E_2_ but not SERMs in CD1 MOE cells (Fig. 3a). This E_2_ dependent proliferative increase was blocked by all the SERMs tested. No E_2_ dependent growth increase was observed in the FVB MOEs suggesting a strain specific E_2_ responsiveness (Additional file 3: Figure S2b).

To determine if E_2_ and SERMs alters the migration of MOE cells, a wound-healing assay was utilized. No difference in migration was observed for either MOE cell line (Fig. 3b and Additional file 3: Figure S2c), suggesting E_2_ does not enhance the migration of normal oviductal epithelial cells.

### RNAseq of MOE cells

The particular set of genes regulated by E_2_ is cell type specific [15]. A number of E_2_-regulated genes in breast cancer cell lines were not altered by E_2_ treatment in MOE cells, for example *Nrip1* (Additional file 3: Figure S2e) [35]. To identify E_2_ regulated genes in the oviduct epithelia, RNAseq was performed on FVB MOE cells treated for 24 h with solvent (DMSO), 1 nM E_2_ or 100 nM 4OHT in triplicate. The FVB MOE cell line was utilized for two reasons. First, HGSC is thought to arise from the secretory cells of the fallopian tube. The FVB MOE line is a monoclonal cell line of exclusively secretory cells [17]. Second, the cell model was chosen instead of primary oviduct culture since primary cells would have contaminating ciliated and underlying stromal cells that could confound the results [36]. Over 438 million reads between the 9 samples (between 40-60 million per sample) were sequenced using Illumina HiSeq2500 platform (Additional file 1: Table S2).

**Fig. 4:**
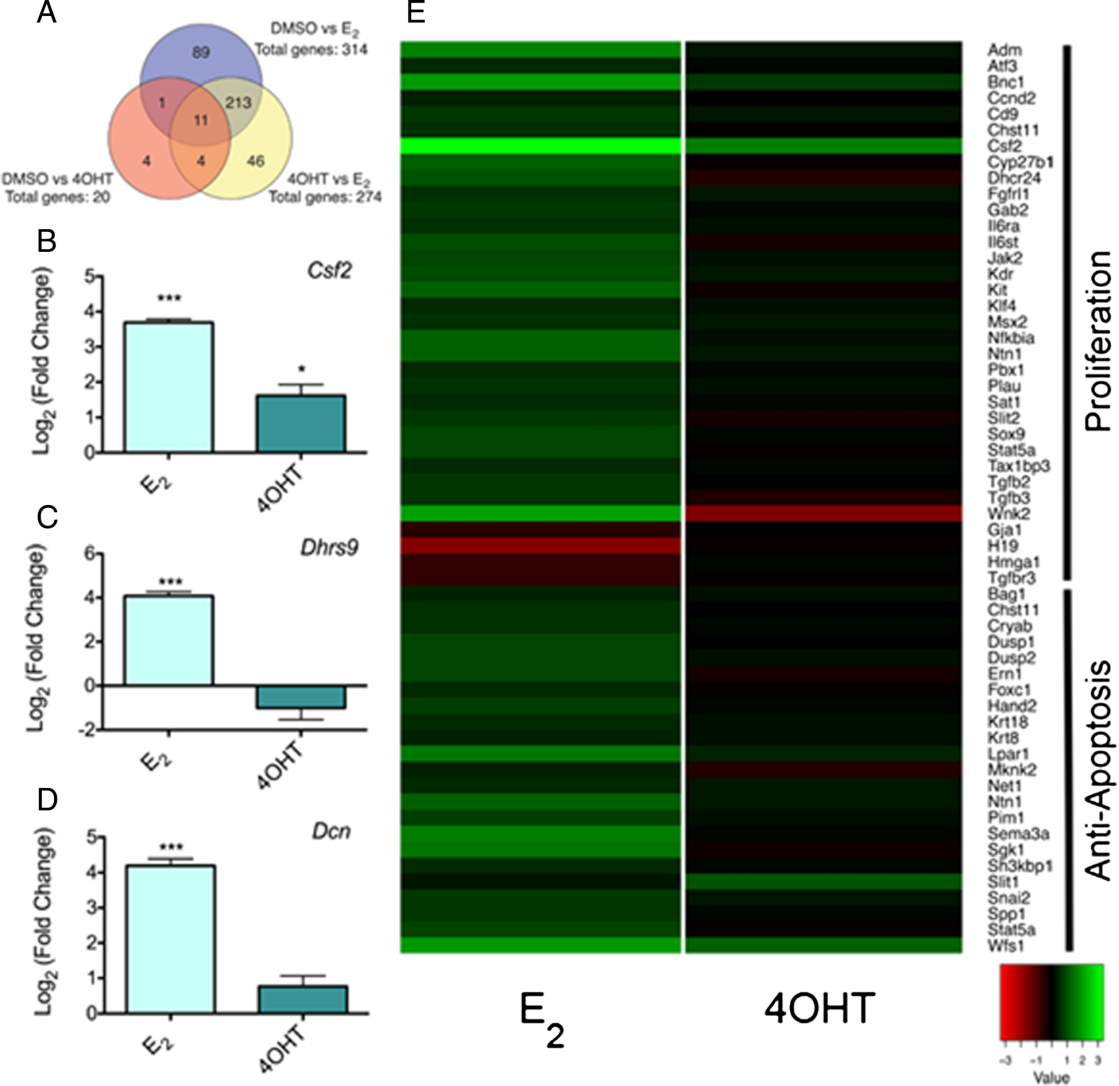
RNAseq analysis of FVB MOE secretory cells treated with E_2_ and 4OHT. **a** Venn diagram of overlap of regulated genes between conditions. **b**-**d** qPCR analysis of genes identified as E_2_ regulated by RNAseq. Significant difference from DMSO control represented by *** (*p* < 0.001) and * (*p* < 0.05) for b) *Csf2*. c) *Dhrs9*. d) *Dcn*. **e** Heat map of genes significantly regulated by E_2_ clustered by GO analysis for biological processes. Log2 Fold change shown for DMSO versus E_2_ and DMSO versus 4OHT

Analysis of the dataset identified 314 gene targets differentially expressed (287 upregulated and 28 downregulated, FDR adjusted *p*-value < 0.05) in response to E_2_ compared to DMSO treated MOE cells. The top 15 genes significantly up and down regulated are listed in Table 1 and 2, respectively. Few genes were shown to be differentially expressed between DMSO and 4OHT (Additional file 1: Table S3 and Additional file 1: Table S4) and only 4 were 4OHT specific, further suggesting that 4OHT functions as an ER antagonist in this cell type. 274 genes targets were differentially expressed between E_2_ and 4OHT treated MOE cells. Figure 4a illustrates the number of regulated genes in common between each condition, highlighting a large overlap in genes differentially regulated by E_2_ compared to DMSO and 4OHT. A subset of genes, *Csf2*, *Dhrs9*, and *Dcn* were validated for response to E_2_ and 4OHT by qPCR (Fig. 4b-d).

**Table 1:** Top 15 up-regulated genes between DMSO and E_2_ treated MOE cells

Gene	Description	Log2 fold change	FDR adjusted *p*-value
*Bpifc*	BPI fold containing family C	4.01	5.31E-03
*Gbp8*	Guanylate-binding protein 8	3.36	5.31E-03
*Csf2*	Colony stimulating factor 2 (granulocyte-macrophage)	3.30	5.31E-03
*Cldn10*	Claudin 10	3.21	5.31E-03
*Ptprz1*	Protein tyrosine phosphatase, receptor type Z, polypeptide 1	3.13	5.31E-03
*Dhrs9*	Dehydrogenase/reductase (SDR family) member 9	3.10	5.31E-03
*Oit1*	Oncoprotein induced transcript 1	2.65	5.31E-03
*Wasf3*	WAS protein family, member 3	2.65	5.31E-03
*Bcas1*	Breast carcinoma amplified sequence 1	2.62	5.31E-03
*Pgr*	Progesterone receptor	2.61	5.31E-03
*Dcn*	Decorin	2.51	5.31E-03
*F5*	Coagulation factor V	2.39	5.31E-03
*Krtap1-5*	Keratin associated protein 1-5	2.33	5.31E-03
*Padi2*	Peptidyl arginine deiminase, type II	2.32	5.31E-03
*Padi1*	Peptidyl arginine deiminase, type I	2.31	5.31E-03

**Table 2:** Top 15 down-regulated genes between DMSO and E_2_ treated MOE cells

Gene	Description	Log2 fold change	FDR adjusted *p*-value
H19	H19, imprinted maternally expressed transcript	-1.65	0.02
Mep1a	Meprin 1 alpha	-1.48	0.03
Rgs8	Regulator of G-protein signaling 8	-1.35	0.05
Hhip	Hedgehog-interacting protein	-1.26	0.01
G6pc2	Glucose-6-phosphatase, catalytic, 2	-1.14	0.01
Adamts16	A disintegrin-like and metallopeptidase (reprolysin type) with thrombospondin type 1 motif, 16	-1.09	0.01
Apln	Apelin	-1.05	0.01
Cyp26a1	Cytochrome P450, family 26, subfamily a, polypeptide 1	-0.88	0.02
Upk3b	Uroplakin 3B	-0.83	0.05
Aqp1	Aquaporin 1	-0.70	0.03
Nup210	Nucleoporin 210	-0.65	0.05
Tgfbr3	Transforming growth factor, beta receptor III	-0.64	0.02
I830012O16 Rik, Ifit3	RIKEN cDNA I830012O16 gene	-0.63	0.01
Havcr1	Hepatitis A virus cellular receptor 1	-0.62	0.01
Pdk4	Pyruvate dehydrogenase kinase, isoenzyme 4	-0.62	0.01

GO analysis of the upregulated genes in response to E_2_ identified a number of biological processes. The heat map shown in Fig. 4e reflect changes in expression between DMSO versus E_2_ or 4OHT for the two most enriched biological processes. Regulation of proliferation was the largest group identified. Regulation of apoptosis was the second highest group enriched. Other significantly enriched processes include response to steroid hormone stimulus, mammary gland epithelium development and calcium ion homeostasis.

### OVCA cell line receptor status

**Fig. 5:**
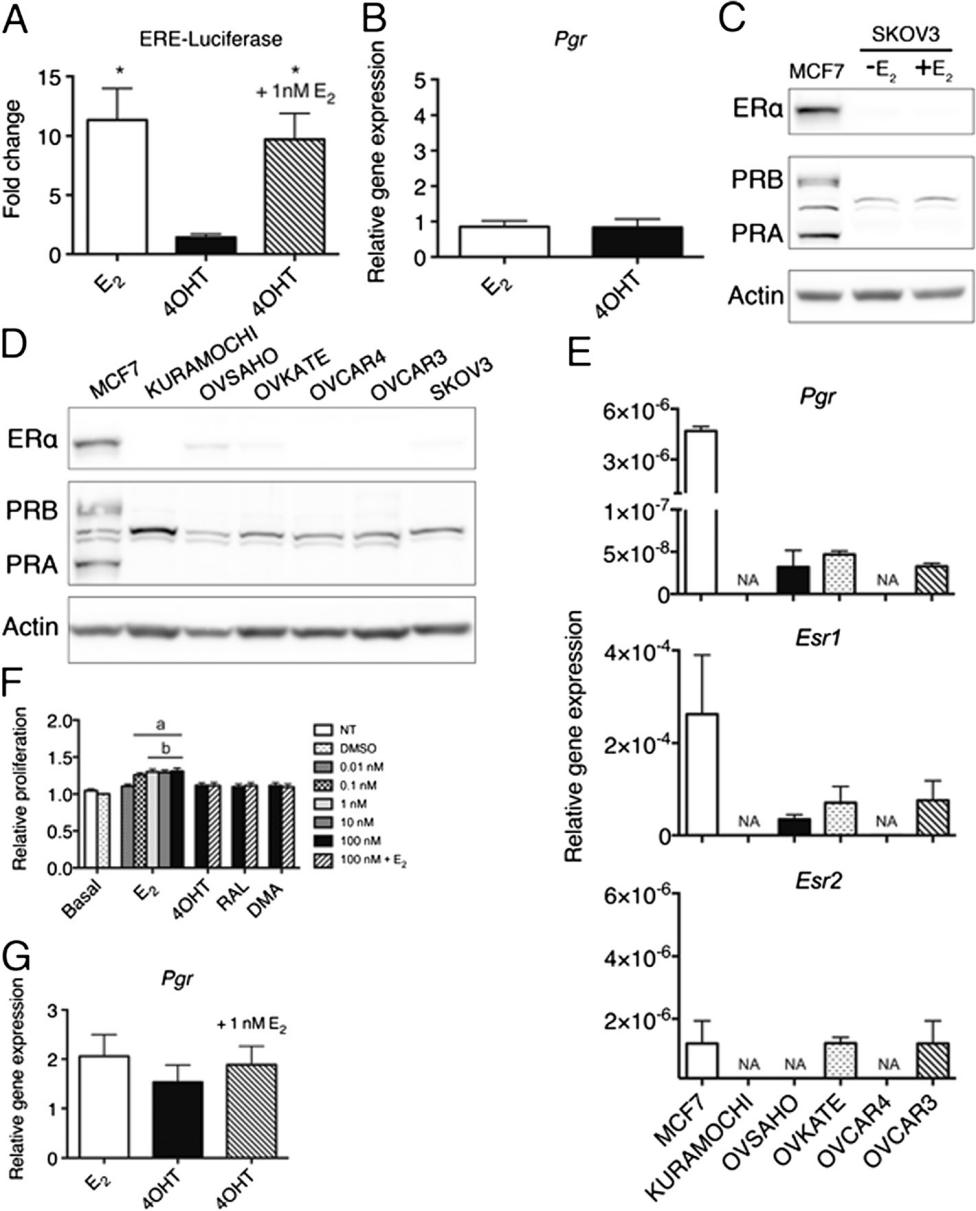
Estrogen signaling in ovarian cancer cell lines. **a** Luciferase reporter assay of SKOV3 cells transfected, hormone starved for 48 h and treated for 24 h with 1 nM E_2_ and or 100 nM 4OHT. ERE-luciferase activity was normalized for transfection efficiency to the betagal control signal. Significant difference from DMSO control denoted by * (*p* < 0.05). **b** qPCR analysis of *Pgr* mRNA levels in SKOV3 as described in **a**. **c** Western blot analysis of ERα and PR levels in SKOV3 cells as described in (**d**) Western blot analysis of ERα and PR status of a panel of HGSC cell lines with positive control MCF7 cells and the commonly used undifferentiated OVCA cell line SKOV3. OVSAHO, OVKATE and SKOV3 reveal low ERα expression, while none of the cell lines expressed detectable PR expression compared to MCF7. **e**-**g** qPCR analysis of *Esr1*, *Pgr* and *Esr2* mRNA levels in HGSC cell lines. **h** Relative growth of OVSAHO cells treated with 1 nM E_2_ or 100 nM SERMs for 7 days. Cells were grown as described in Fig. 3a except media was replenished after 72 h. Significant difference from untreated and DMSO treated cells represented by “a” (*p* < 0.01) and significant difference from SERM treated cells represented by “b” (*p* < 0.05) i) qPCR analysis of *Pgr* induction in response to E_2_ and 4OHT treatment in OVSAHO cells

Estrogen only hormone replacement therapy and the number of lifetime ovulations are associated with OVCA [8] . The SKOV3 cell line is the most cited OVCA cell line [37]. Previous reports indicate that SKOV3 are not growth responsive to E_2_ due to expression of a truncated ERα [38]. While SKOV3 were responsive to E_2_ by a reporter assay containing an ERE-binding element fused to a luciferase gene (Fig. 5a), they were not at the endogenously regulated *PGR* gene by either qPCR or Western blot analysis (Figs. 5b, c). Another commonly used E_2_-responsive cell line in the OVCA literature, BG1, was reported to proliferate in response to estrogen, although a subset of studies using this cell line has subsequently been identified as MCF7 breast cancer cells [39]. Currently, neither cell type are genomically validated models of HGSC, thought to develop from fallopian tube cells [37] and highlight the need to explore additional cell models.

We analyzed a number of representative and commercially available HGSC lines for the presence of the steroid hormone receptors ERα, PR, and ERβ. Molecular profiling of these cell lines predict OVSAHO, Kuramochi, OVCAR4 and OVKATE are likely, while OVCAR3 is possibly and SKOV3 is unlikely to be HGSC [37]. All OVCA cell lines tested expressed much less or no ERα and PR compared to the receptor positive breast cancer cell line MCF7 by both Western blot and RNA analyses (Fig. 5d-e). ERβ status was monitored for all samples by qPCR revealing minimally detectable amounts mRNA (Fig. 5e, a cell line expressing a plasmid encoding ERβ was used to validate the primers). The dearth of ER and PR receptors in OVCA cell lines is in contrast to the 60 % of ER positive tumors [13] and makes investigation into the role of estrogen in OVCA challenging.

Despite low levels of ERα and PR in the OVCA cell lines, one HGSC cell line, OVSAHO, which expressed ERα and PR was tested for E_2_ responsiveness. OVSAHO proliferation was significantly increased by E_2,_ but not SERMs, following a 7 day treatment, and the SERMs antagonized E_2_ dependent growth (Fig. 5f). Following 24-hour treatment, a mild albeit not significant induction was observed at the *PGR* gene in response to E_2_, 4OHT and E_2_ plus 4OHT (Fig. 5g). Therefore, despite the lower levels of receptors compared to MCF7 breast cancer cells, OVSAHO are an estrogen responsive cell model.

## Discussion

The epithelium of the fallopian tube is considered to be one of the origins of HGSC and estrogen replacement therapy impacts the risk of OVCA. This study characterized the agonist/antagonist function of SERMs in a normal murine model of fallopian tube epithelium, identified the genes regulated by estrogen signaling in this cell type to shed light on the subset of genes regulated by E_2_ in this cell type, and determined the E_2_ responsiveness of a panel of HGSC cell lines.

### SERMS antagonize E_2_ in MOE cells

Tamoxifen is a common treatment for receptor positive breast cancer, as well as given prophylactically for women at high risk for developing breast cancer [31]. Given the risk associated with Tamoxifen for the uterus, the response of Tamoxifen and other SERMs in the fallopian tube epithelium is highly important for clarifying the risks of using these drugs in women with intact fallopian tubes. Responses such as proliferation or migration has not been well documented in fallopian tube epithelium as compared to the response in the breast or uterine tissue, traditional hormone responsive tumor types [20]. Reports from our lab indicate that estrogen did not proliferate oviductal epithelium in FVB strains, or in organotypic cultures of the mouse, baboon, or human [20, 40]. Long term (at least 4 years) Tamoxifen treatment of women with breast cancer has been reported to increase tubal dysplasia [41]. The anti-estrogen effect reported here on isolated epithelial cell lines suggests the dysplastic effect of tamoxifen may not be due to proliferation, but rather the length of exposure, or a non transcriptional effects, such as DNA damage [42].

### RNAseq identifies targets of E_2_ signaling in MOE cells

Many E_2_ regulated genes in other normal and cancer cell types were also regulated in MOE cells including *Pgr*, *Greb1*, *Csf2*, and *Dhrs9* [43, 44], while other E_2_ regulated genes in MCF7 cells were not regulated (*Nrip1*) [35] or even expressed (*Tff1*) in MOE cells [45]. Interestingly, in contrast to MCF7 [45], the majority of differentially expressed genes were upregulated in MOE cells at the time point (24 h) probed. Table 1 and 2 lists the most highly regulated genes in response to E_2_ in MOE cells, some of which are relatively uncharacterized. BPI fold containing family C gene (*Bpifc*), involved in lipid binding [46], demonstrated the largest induction in response to E_2_. One gene specifically upregulated by 4OHT was the RNA component of mitochondrial processing endonuclease (*Rmrp)*, reported to be upregulated in 4OHT resistant as compared to 4OHT sensitive breast cancer cells [47]. The biological processes enriched in MOE cells included regulation of proliferation and apoptosis, response to hormone stimulus and calcium ion homeostasis. The RNAseq analysis identified genes responsible for both positive and negative regulation of proliferation, which may reconcile the lack of significant proliferative increase in the FVB MOE background. In the fallopian tube, calcium is required for sperm capacitation [48] and cilia beating [49], therefore E_2_ may regulate calcium levels in the fallopian tube as part of reproductive biology.

Comparison of the MOE E_2_ responsive genes and The Cancer Genome Atlas of ovarian cancer tumors identified a number of genes with significance in OVCA in common between the two groups [27, 50, 51]. For example, E_2_ increased expression of the cyclin dependent kinase 2 (*Ccnd2*) gene, which is altered (via amplification or mRNA upregulation) in 12 % of ovarian tumors, and has been shown to be upregulated in some OVCAs [52]. *Jak2*, and *Kit* were also upregulated in response to E_2_ and altered in 12 % and 8 % of ovarian tumors, respectively. The *St3gal1* gene, encoding a glycosyltransferase, was upregulated by E_2_ and altered (mostly amplified or showing mRNA upregulation) in 30 % of ovarian tumors. The significance of ST3Gal1 in OVCA is unknown, but has been linked to colon cancer [53]. Cyclin dependent kinase 1 (*Ccnd1*) was downregulated in response to E_2_ and altered in 8 % of ovarian tumors. Interestingly, CCND1 is overexpressed in cisplatin resistant testicular cancer and OVCA [54]. The overlap of genes regulated by E_2_ in MOE cells and alteration in OVCA provides a number of potential new targets for further investigation of E_2_ regulation in OVCA.

### Need for better E_2_ responsive models of HGSC

The most frequently used estrogen responsive OVCA cell lines are not ideal models of HGSC including SKOV3 and the NIEHs BG1 cells [38, 39]. Two other estrogen responsive OVCA cell lines, PEO1 and PEO4, have recently been reported as HGSC [55]. These cell lines proliferate in response to E_2_ in culture and xenografts and E_2_ increases risk of distant metastases [32, 33]. The HGSC cell lines investigated in this study express much less ER and PR receptors compared to the estrogen responsive MCF7. Nevertheless, the likely HGSC cell line OVSAHO proliferates in response to E_2_, but not SERMs. Further validation of other/more estrogen responsive HGSC cell lines is desperately needed to aid in understanding the role of estrogen in OVCA and whether ER expressing HGSC would respond to anti-estrogen therapy. By studying how the cancers and different progenitors, such as the oviductal or OSE, respond to estrogen may aid in the use of SERMs in tumors that express ER or help to uncover if long-term use of Tamoxifen could enhance dysplastic lesions in the fallopian tube.

## Conclusion

This study shows that the fallopian tube epithelia respond to E_2_ stimulation by regulating expression of a tissue-specific set of target genes. All SERMs tested inhibited E_2_-stimulated responses, showing SERMs are antagonistic action in the fallopian tube. Thus direct effects of E_2_ on the fallopian tube epithelium may play a role in the development of ovarian cancer.

## Ethics approval

Not Applicable.

## Consent for publication

Not applicable.

## Availability of data and material

The sequences and expression values reported in this paper have been deposited in the NCBI GEO database, Accession number GSE67207.

## Additional files

Additional file 1:
**Table S1.** Primers used in study. **Table S2.** RNAseq read counts. **Table S3.** Genes significantly up-regulated by 4OHT in MOE cells. **Table S4.** Genes significantly down-regulated by 4OHT in MOE cells. (DOC 78 kb) Additional file 2: Figure S1. E_2_ response in putative OVCA precursor cell types. a, b) Densitometry of ERα and PRA protein levels in response to 1 nM E_2_ treatment (relative to solvent control and normalized to actin) of FVB MOE and FVB MOEp53^R273H^ cells. c) qPCR analysis of *Pgr* induction in response to 24 h treatment with 1 nM E_2_ in FVB MOE and FVB MOEp53^R273H^ cells. d) FVB MOSE cells treated with an HDAC inhibitor (HD13) does not upregulate ERα or recover E_2_ responsiveness. Significant difference relative to DMSO control denoted by * (*p* < 0.05), ** (*p* < 0.01), and *** (*p* < 0.001). (TIF 150 kb) Additional file 3: Figure S2. FVB MOE response to E_2_ and SERMs. a, b) qPCR analysis of (a) *Pgr* and (b) *Greb1* induction in response to 48 h hormone starvation and 24 h treatment with solvent, 1 nM E_2_ or 100 nM SERMs. “a” indicates significant difference compared to all treatments. c) Relative growth of FVB MOE following 72-hour hormone starvation and 72-hour treatment with solvent, 1 nM E_2_, 100 nM SERMs and the combination as monitored by SRB assay. d) Relative migration of FVB MOE cells following 72-hour hormone starvation and 24-hour treatment with solvent, 1 nM E_2_ or 100 nM SERMs. Wound initiated at time of treatment. e) qPCR analysis of Nrip1 expression levels in CD1 and FVB MOE cells in response to 24 h treatment with DMSO or 1 nM E_2_. (TIF 615 kb)

## Abbreviation

DMA: desmethylarzoxifene
E_2_: Estrogen
ER: estrogen receptor
FTSEC: fallopian tube secretory epithelial cell
HGSC: high-grade serous ovarian cancer
MOE: murine oviductal epithelium
MOSE: murine ovarian surface epithelium
OSE: ovarian surface epithelium
OVCA: ovarian cancer
PR: progesterone receptor
RAL: raloxifene
SERMs: selective estrogen receptor modulators
4OHT: 4-hydroxytamoxifen
