# Supplementary material for: Genome-wide transcriptional regulation of estrogen receptor targets in fallopian tube cells and the role of selective estrogen receptor modulators
Source: J Ovarian Res. 2016 Feb 15;9:5. doi: 10.1186/s13048-016-0213-3 (PMC4754840; doi:10.1186/s13048-016-0213-3)
Supplement: Additional file 1: — Table S1. Primers used in study. Table S2. RNAseq read counts. Table S3. Genes significantly up-regulated by 4OHT in MOE cells. Table S4. Genes significantly down-regulated by 4OHT in MOE cells. (DOC 78 kb) [file 13048_2016_213_MOESM1_ESM.doc]

**Tables**

| Supplemental Table 1: Primers used in study | | | |
| --- | --- | --- | --- |
| Gene (mouse) | Forward Primer | Reverse Primer |  |
| *18S* | TCAACTTTCGATGGTAGTCGCCGT | TCCTTGGATGTGGTAGCCGTTTCT |  |
| *Bcas1* | AGATACTCCCACCGACTTAC | CATTGTCTGACGTCACCTT |  |
| *Csf2* | AAGATATTCGAGCAGGGTCTA | AGTCCGTTTCCGGAGTT |  |
| *Dcn* | CTGGCCAATGTTCCTCATC | GGTAGACGACCTGGATATACTT |  |
| *Dhrs9* | CCTAAGGCGGGATATGAAAG | TCAGTTGTCTTGATTGGATCTG |  |
| *Greb1* | ATGCCCTGCTTGGTTTC | CTTGAGGTGCTTCTGTTTCT |  |
| *Nrip1* | CTTCCTTTCCCACATAGCAG | GCAACTTCCTTAGCACAGC |  |
| *Pgr* | TCTACCCGCCATACCTTAAC | CCTTCCATTGCCCTCTTAAAG |  |
| Gene (human) |  |  |  |
| *18S* | GCTTGCGTTGATTAAGTCCC | GCCTCACTAAACCATCCAATC |  |
| *ESR1* | TCCTACCAGACCCTTCAGTG | GGTCAAATCCACAAAGCCTG |  |
| *ESR2* | CCTTACCTGTAAACAGAGAGAC | CTTTGAACCTGGACCAGTAA |  |
| *PGR* | TTTAAGAGGGCAATGGAAGG | CGGATTTTATCAACGATGCAG |  |

| *Supplemental Table 2: RNAseq read counts* | | | | |
| --- | --- | --- | --- | --- |
| Sample | Treatment | Initial Reads | rRNA Removal | Trimming Adapters |
| GH1 | DMSO | 50,305,520 | 49,267,348 | 49,265,743 |
| GH2 | E_2_ | 43,585,248 | 42,673,119 | 42,672,069 |
| GH3 | 4OHT | 45,846,656 | 45,032,785 | 45,031,940 |
| GH4 | DMSO | 60,505,583 | 59,473,164 | 59,471,821 |
| GH5 | E_2_ | 48,832,405 | 48,044,924 | 48,042,237 |
| GH6 | 4OHT | 41,812,551 | 41,116,107 | 41,115,150 |
| GH7 | DMSO | 53,509,644 | 52,600,499 | 52,598,451 |
| GH8 | E_2_ | 54,747,034 | 53,816,236 | 53,814,207 |
| GH9 | 4OHT | 47,365,720 | 46,221,316 | 46,220,162 |

| Supplemental Table 3: Genes significantly up-regulated by 4OHT in MOE cells | | | |
| --- | --- | --- | --- |
| Gene | Description | Log2 Fold Change | FDR adjusted p-value |
| *Csf2* | colony stimulating factor 2 (granulocyte-macrophage) | 1.55 | 0.01 |
| *Rmrp* | RNA component of mitochondrial RNAase P | 1.30 | 0.01 |
| *Rpph1* | ribonuclease P RNA component H1 | 1.04 | 0.05 |
| *Padi2* | peptidyl arginine deiminase, type II | 1.02 | 0.01 |
| *Tmem20* | solute carrier family 35, member G1 | 0.72 | 0.01 |
| *Padi1* | peptidyl arginine deiminase, type I | 0.65 | 0.01 |
| *Hsd17b11* | hydroxysteroid (17-beta) dehydrogenase 11 | 0.52 | 0.03 |

| Supplemental Table 4: Genes significantly down-regulated by 4OHT in MOE cells | | | |
| --- | --- | --- | --- |
| Gene | Description | Log2 Fold Change | FDR adjusted  p-value |
| *Spaca6* | sperm acrosome associated 6 | -1.58 | 0.005 |
| *Gbp9* | guanylate-binding protein 9 | -1.56 | 0.005 |
| *Wnk2* | WNK lysine deficient protein kinase 2 | -1.55 | 0.005 |
| *B3gnt8* | UDP-GlcNAc:betaGal beta-1,3-N-acetylglucosaminyltransferase 8 | -1.33 | 0.005 |
| *Dhrs9* | dehydrogenase/reductase (SDR family) member 9 | -1.24 | 0.005 |
| *Adamts16* | a disintegrin-like and metallopeptidase (reprolysin type) with thrombospondin type 1 motif, 16 | -1.09 | 0.005 |
| *Sema3g* | sema domain, immunoglobulin domain (Ig), short basic domain, secreted, (semaphorin) 3G | -0.95 | 0.022 |
| *BC005561,*  *D930016D06Rik* | RIKEN cDNA D930016D06 gene | -0.61 | 0.045 |
| *Mylk* | myosin, light polypeptide kinase | -0.55 | 0.042 |
| *Dhcr24* | 24-dehydrocholesterol reductase | -0.48 | 0.025 |
| *Kcnq1ot1* | KCNQ1 overlapping transcript 1 | -0.47 | 0.032 |
| *Fat1* | FAT tumor suppressor homolog 1 (Drosophila) | -0.45 | 0.042 |
| *Neat1* | nuclear paraspeckle assembly transcript 1 (non-protein coding) | -0.44 | 0.035 |
| *Lrp2* | low density lipoprotein receptor-related protein 2 | -0.44 | 0.042 |
| *Spaca6* | sperm acrosome associated 6 | -1.58 | 0.005 |

**Supplemental References**

1. Collins F, MacPherson S, Brown P, Bombail V, Williams AR, Anderson RA, Jabbour HN, Saunders PT. Expression of oestrogen receptors, ERalpha, ERbeta, and ERbeta variants, in endometrial cancers and evidence that prostaglandin F may play a role in regulating expression of ERalpha. BMC Cancer 2009; 9:330.
