# Supplementary figures and images for: Genome-wide transcriptional regulation of estrogen receptor targets in fallopian tube cells and the role of selective estrogen receptor modulators
Source: J Ovarian Res. 2016 Feb 15;9:5. doi: 10.1186/s13048-016-0213-3 (PMC4754840; doi:10.1186/s13048-016-0213-3)

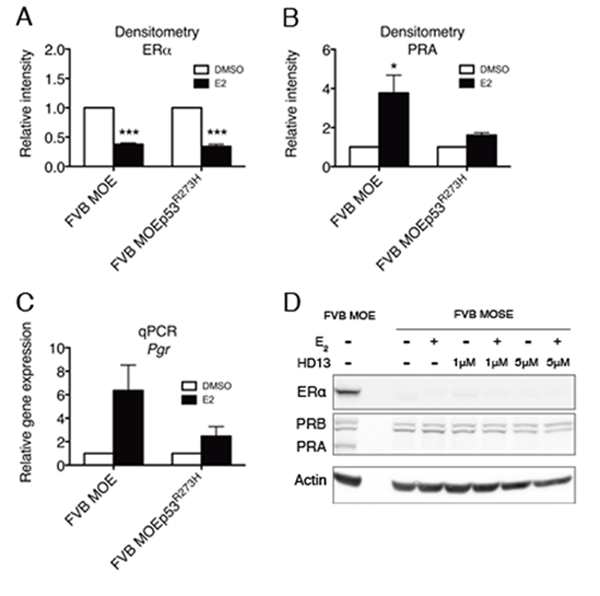

Supplement: Additional file 2: Figure S1. — E2 response in putative OVCA precursor cell types. a, b) Densitometry of ERα and PRA protein levels in response to 1 nM E2 treatment (relative to solvent control and normalized to actin) of FVB MOE and FVB MOEp53R273H cells. c) qPCR analysis of Pgr induction in response to 24 h treatment with 1 nM E2 in FVB MOE and FVB MOEp53R273H cells. d) FVB MOSE cells treated with an HDAC inhibitor (HD13) does not upregulate ERα or recover E2 responsiveness. Significant difference relative to DMSO control denoted by * (p < 0.05), ** (p < 0.01), and *** (p < 0.001). (TIF 150 kb) [file 13048_2016_213_MOESM2_ESM.tif]

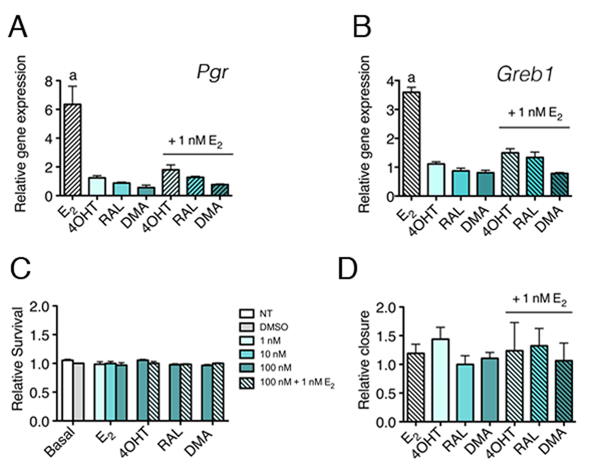

Supplement: Additional file 3: Figure S2. — FVB MOE response to E2 and SERMs. a, b) qPCR analysis of (a) Pgr and (b) Greb1 induction in response to 48 h hormone starvation and 24 h treatment with solvent, 1 nM E2 or 100 nM SERMs. “a” indicates significant difference compared to all treatments. c) Relative growth of FVB MOE following 72-hour hormone starvation and 72-hour treatment with solvent, 1 nM E2, 100 nM SERMs and the combination as monitored by SRB assay. d) Relative migration of FVB MOE cells following 72-hour hormone starvation and 24-hour treatment with solvent, 1 nM E2 or 100 nM SERMs. Wound initiated at time of treatment. e) qPCR analysis of Nrip1 expression levels in CD1 and FVB MOE cells in response to 24 h treatment with DMSO or 1 nM E2. (TIF 615 kb) [file 13048_2016_213_MOESM3_ESM.tif]
